# Intracellular fate of *Ureaplasma parvum* entrapped by host cellular autophagy

**DOI:** 10.1002/mbo3.441

**Published:** 2017-01-15

**Authors:** Fumiko Nishiumi, Michinaga Ogawa, Yukiko Nakura, Yusuke Hamada, Masahiro Nakayama, Jiro Mitobe, Atsushi Hiraide, Norio Sakai, Makoto Takeuchi, Tamotsu Yoshimori, Itaru Yanagihara

**Affiliations:** ^1^ Department of Developmental MedicineOsaka Medical Center and Research Institute for Maternal and Child HealthOsakaJapan; ^2^ Department of Bacteriology INational Institute of Infectious DiseasesTokyoJapan; ^3^ Department of PediatricsOsaka University Graduate School of MedicineOsakaJapan; ^4^ Department of PathologyOsaka Medical Center and Research Institute for Maternal and Child HealthOsakaJapan; ^5^ Critical Care Medical CenterFaculty of MedicineKindai UniversityOsakaJapan; ^6^ Division of Health ScienceOsaka University Graduate School of MedicineOsakaJapan; ^7^ Department of GeneticsGraduate School of MedicineOsaka UniversityOsakaJapan

**Keywords:** autophagy, endocytosis, exosome, galectin, *Ureaplasma* spp

## Abstract

Genital mycoplasmas, including *Ureaplasma* spp., are among the smallest human pathogenic bacteria and are associated with preterm birth. Electron microscopic observation of *U. parvum* showed that these prokaryotes have a regular, spherical shape with a mean diameter of 146 nm. *U. parvum* was internalized into HeLa cells by clathrin‐mediated endocytosis and survived for at least 14 days around the perinuclear region. Intracellular *U. parvum* reached endosomes in HeLa cells labeled with EEA1, Rab7, and LAMP‐1 within 1 to 3 hr. After 3 hr of infection, *U. parvum* induced the cytosolic accumulation of galectin‐3 and was subsequently entrapped by the autophagy marker LC3. However, when using *atg7* ^−/−^ MEF cells, autophagy was inadequate for the complete elimination of *U. parvum* in HeLa cells*. U. parvum* also colocalized with the recycling endosome marker Rab11. Furthermore, the exosomes purified from infected HeLa cell culture medium included *U. parvum*. In these purified exosomes ureaplasma lipoprotein multiple banded antigen, host cellular annexin A2, CD9, and CD63 were detected. This research has successfully shown that *Ureaplasma* spp. utilize the host cellular membrane compartments possibly to evade the host immune system.

## 1 INTRODUCTION

Phylogenetically, mycoplasmas are probably the latest product of evolution among bacteria resulting from genome reduction (also known as regressive evolution). These organisms have limited metabolic pathways for replication and depend on the host for the supply of exogenous membrane components including fatty acids, cholesterol, and complex lipids for survival (Kornspan & Rottem, 2012). These organisms are bound by a plasma membrane and lack rigid cell walls. Mycoplasmas have mainly been isolated from the mucosal surfaces (Volgmann, Ohlinger, & Panzig, 2005; Waites, Schelonka, Xiao, Grigsby, & Novy, 2009; Yi, Yoon, & Kim, 2005) and some have been reported to be internalized into the host cells (Marques et al., 2010; Winner, Rosengarten, & Citti, 2000; Yavlovich, Katzenell, Tarshis, Higazi, & Rottem, 2004). *Ureaplasma* spp. belong to the family *Mycoplasmataceae*. These are the smallest self‐replicating organisms in terms of genome size and cellular dimensions. *Ureaplasma parvum* (*U. parvum*) and *U. urealyticum*, which are common inhabitants of the human lower genital tract, have been isolated from 40% to 80% of women among child‐bearing age (Taylor‐Robinson & McCormack, 1980). However, when *Ureaplasma* spp. spread in the genital tract during gestation, they have the potential to be pathogens and cause chorioamnionitis, resulting in spontaneous abortion or preterm birth (Namba et al., 2010).

The mechanisms by which viruses and bacteria are internalized into host cells are known mainly to involve two pathways, fusion and endocytosis. The endocytotic pathways exploited by animal viruses to gain entry into host cells include macropinocytosis, clathrin‐dependent endocytosis, and caveolae‐dependent endocytosis. Rab proteins are involved in various aspects of endocytic and exocytic protein transport through their specific association with membrane vesicles or organelles. Early endosome antigen 1 (EEA1) is a major marker of the early endosome stage (Christoforidis, McBride, Burgoyne, & Zerial, 1999; Simonsen et al., 1998). After this stage, the phagosome loses the marker associated with early endosome, Rab5, and acquires Rab7 and markers associated with the late endosome stage such as a transmembrane protein enriched in late endosomes and lysosome‐associated membrane protein 1 (LAMP‐1) (Desjardins, 1995; Desjardins, Huber, Parton, & Griffiths, 1994; Pitt, Mayorga, Schwartz, & Stahl, 1992). The eukaryotic cytoskeleton is targeted by a variety of bacterial pathogens during the course of infection and dynamic changes of the cytoskeleton influence the interaction of microbial pathogens with the host cells. Microbial pathogens deliver a number of effector proteins to the host cells to rearrange the cytoskeleton in a way that promotes infection. Many bacterial pathogens modulate microtubule dynamics by employing virulence proteins to promote infection (Radhakrishnan & Splitter, 2012). Intact microtubules are also essential for other polyomaviruses, including SV40 (Pelkmans, Kartenbeck, & Helenius, 2001).

Galectins are beta‐galactoside‐binding lectins that accumulate in the cytosol before being secreted via a leader peptide‐independent pathway (Houzelstein et al., 2004; Rabinovich & Toscano, 2009). During an infection, galectin‐3 was suggested to be a potential receptor for pathogen recognition based on its ability to bind certain bacterial, parasitic, and fungal products (Sato & Nieminen, 2004). Galectin‐3 was also proposed to be a potential immunological danger signal based on its passive release from cells at the site of infection and its active release from inflammatory macrophages (Liu & Hsu, 2007; McClung et al., 2007; Sato & Nieminen, 2004). The accumulation of galectin‐3 in host cells is known to induce autophagy (Chen, Weng, Hong, & Liu, 2014).

Autophagy protects host cells from pathogenic bacteria (Birmingham, Smith, Bakowski, Yoshimori, & Brumell, 2006; Fujita et al., 2009; Sun et al., 2008). In mammals, *Atg7* was shown to be essential for ATG12 conjugation, microtubule‐associated protein 1 light chain 3 (LC3) modification systems, and autophagosome formation. *Atg7* mutant mice should be useful for examining the role of autophagy in the cell death pathway or in a cellular defense mechanism in the pathogenesis of these diseases (Komatsu et al., 2005). LC3 is the first mammalian protein localized in the autophagosome membrane. A portion of invading *Salmonella typhimurium* is associated with LC3 (Birmingham et al., 2006; Kabeya et al., 2000; Kageyama et al., 2011). However, the autophagic machinery may play a role in establishing resident bacteria. These processes are collectively called *xenophagy* and the molecular mechanisms that govern these processes are only now beginning to be analyzed (Deretic & Levine, 2009; Huang & Brumell, 2009; Levine, 2005; Noda & Yoshimori, 2009).

It was reported that the genital mycoplasmas *Ureaplasma* spp. and *Mycoplasma hominis* were detected in the cord blood of 23% of preterm birth babies (Goldenberg et al., 2008). This report also indicated that fetal *U. parvum* infection is caused not only by ascending infection from the lower genital tract but also by hematogenous vertical transmission; nevertheless, the mechanism underlying feto‐maternal transmission is still unknown. In this report, we reveal the mechanisms of internalization and intracellular survival of *U. parvum* in HeLa cells and present the possibility of host cellular exosome‐mediated transmission of bacteria. Such exosome‐mediated transmission may facilitate escape from the human immune system and may contribute to the feto‐maternal transmission of *U. parvum*.

## 2 EXPERIMENTAL PROCEDURES

### 2.1 The clinical specimen and *U. parvum* strains

All clinical specimens were obtained after receiving informed consent and approval from the Ethics Committee of Osaka Medical Center and Research Institute for Maternal and Child Health. For the pathological examinations, placenta from preterm delivery at 29 weeks of gestation (Namba et al., 2010) was used. This placenta was culture‐positive for *U*. *parvum*, which was determined by analysis of the DNA sequence of the 16S rRNA gene (data not shown). The sequence primers used were as follows: (27f: 5′‐AGAGTTTGATCCTGGCTCAG‐3′, 1525r: 5′‐AAAGGAGGTGATCCAGCC‐3′). For in vitro infection studies, we used clinical isolates of the *U. parvum* serovar 3 strain derived from human placenta of a preterm delivery at 26 weeks of gestation [*U. parvum* OMC‐P162 (Uchida et al., 2013)].

### 2.2 Immunohistochemistry

Paraffin‐embedded sections of human placenta with *Ureaplasma* spp. were deparaffinized and rehydrated. The sections were incubated with rabbit anti‐MBA polyclonal antibody (1:500) against the common N‐terminal peptide of multiple banded antigen (MBA) (Namba et al., 2010), which is reproduced by ureaplasmas (Uchida et al., 2013). Immunoreactivity with horseradish peroxidase‐conjugated labeled polymer was detected using the Envision^TM^ + Dual Link System‐HRP (Dako, Carpinteria, CA, USA). Mayer's hematoxylin staining (Muto Pure Chemicals, Tokyo, Japan) was also performed for light microscopy (BX51; Olympus, Tokyo, Japan) evaluation. The study was approved by the Institutional Human Ethical Committee and recommended guidelines were followed during sample collection.

### 2.3 Transmission electron microscopy and pre‐embedding immunoelectron microscopy

Transmission electron microscopy was used to confirm the presence of *U. parvum* in HeLa cells after 1, 3, and 6 hr of infection, and in uninfected control HeLa cells. For ultrastructural analyses, HeLa cells washed with phosphate‐buffered saline (PBS, pH 7.4) were fixed for 15 min in 4% paraformaldehyde (Wako Pure Chemical Industries Ltd., Osaka, Japan) and 0.05% glutaraldehyde in 30 mM HEPES‐HCl, pH 7.3. The cells were washed several times with 100 mM phosphate buffer, 70 mM sucrose (pH 7.4), and postfixed with 2% osmium tetroxide (OsO_4_; Taab Laboratories Equipment Ltd., Reading, UK) in 100 mM phosphate buffer, pH 7.4, for 1 hr at 4°C, dehydrated in a graded series of ethanol, and embedded in epoxy resin (Quetol 812; Nisshin EM, Tokyo, Japan). Ultrathin sections were cut with an ultramicrotome (Ultracut UCT; Leica, Vienna, Austria) at 80 nm thickness, put on a copper grid, stained with 3% uranyl acetate followed by stable lead (Hanaichi et al., 1986), and observed by transmission electron microscopy (TEM) (HT‐7700; Hitachi, Tokyo, Japan).

### 2.4 Scanning electron microscopy

*Ureaplasma parvum* was grown on a UMCH agar plate (Namba et al., 2010) and fixed with 2% glutaraldehyde (Wako) overnight before being cut into small pieces. The samples were then washed, resuspended in PBS, postfixed with 1% osmium tetroxide, and dehydrated with a graded ethanol series. We then conducted a t‐butanol drying process and coated the samples with platinum/palladium by ion sputter E‐1030 (Hitachi). Microscopy was performed with an SU3500 SEM (Hitachi).

### 2.5 Real‐time quantitative polymerase chain reaction (qPCR)

Total DNA from *U. parvum*‐infected (3, 7, 10, and 14 days) HeLa cells was isolated using a QIAmp^®^ DNA Mini kit (Qiagen, Valencia, CA, USA), following the manufacturer's instructions. *U. parvum* DNA expression levels of infected HeLa cells were measured by real‐time qPCR using a QuantiTect SYBR Green PCR kit (Qiagen) and detected using the DNA Engine Opticon^TM^ System (Bio‐Rad, Hercules, CA, USA). The primer sequences were as follows: UreB28F (Fw; 5′‐CCAGGTAAATTAGTACCAGG‐3′), UreB260R (Rv; 5′‐CCTGATGGAATATCGAAACG‐3′).

### 2.6 Plasmid construction

The expression clones pEXPR‐P_EF1α_‐EGFP‐Rab7, pEXPR‐P_EF1α_‐EGFP‐galectin‐1, pEXPR‐P_EF1α_‐mCherry‐galectin‐3, pEXPR‐P_EF1α_‐EGFP‐galectin‐8, pEXPR‐P_EF1α_‐EGFP‐galectin‐9s, and pEXPR‐P_EF1α_‐Annexin A2‐EGFP were constructed by LR reaction with the Destination vector, pEF1/B2B1/V5‐DEST and four types of Entry clone (Invitrogen, Carlsbad, CA, USA), in accordance with the manufacturer's instructions. pENTR‐L1‐sdk‐mCherry‐R3, pENTR‐L1‐sdk‐EGFP‐L4, pENTR‐L1‐sdk‐annexin A2‐L4, pENTR‐R4‐Rab7‐*L2, pENTR‐L3‐galectin‐3‐*L2, pENTER‐L3‐galectin‐1‐*L2, pENTR‐L3‐galectin‐8‐*L2, pENTR‐L3‐galectin‐9s‐*L2, and pENTR‐R4‐EGFP‐*L2 were constructed using the MultiSite Gateway^®^ cloning of attB‐PCR fragments amplified from cDNA plasmids of Rab7 (GenBank accession no. X93499). Construction of the Entry clones of EGFP and Venus was described previously (Sone et al., 2008). The PCR primers used were as follows: Rab7 [forward (FW): 5′‐GGGGACAACTTTTCTATACAAAGTTGATGACCTCTAGGAAGAAA‐3′, reverse (RV): 5′‐GGGGACCACTTTGTACAAGAAAGCTGTCAGCAACTGCAGCTTTC‐3′; galectin‐1 FW: 5′‐GGGGACAACTTTGTATAATAAAGTTGATGGCTTGTGGTCTGGTC‐3′, RV: 5′GGGGACCACTTTGTACAAGAAAGCTGTCAGTCAAAGGCCACACA‐3′; galectin‐3 FW: 5′‐GGGGACAACTTTGTATAATAAAGTTGATGGCAGACAATTTTTCG‐3′, RV: 5′‐GGGGACCACTTTGTACAAGAAAGCTGTTATATCATGGTATATGA‐3′; galectin‐8 FW: 5′‐GGGGACAACTTTGTATAATAAAGTTGATGATGTTGTCCTTAAACAACC‐3′, RV: 5′‐GGGGACCACTTTGTACAAGAAAGCTGCTACCAGCTCCTTACTTCC‐3′; galectin‐9s FW: 5′‐GGGGACAACTTTGTATAATAAAGTTGATGGCCTTCAGCGGTTCC‐3′, RV: 5′‐GGGGACCACTTTGTACAAGAAAGCTGCTATGTCTGCACATGGGT‐3′; annexin A2 FW: 5′‐GCTTCGAAGGAGATAGAACCATGTCTACTGTTCACG‐3′, RV: 5′‐GGGGACAACTTTGTATAGAAAAGTTGGTCATCTCCACCACACAGG‐3′].

### 2.7 Cell culture and generation of stable transformants

HeLa, MEFs, and Atg7 knockout MEFs (*atg7* ^−/−^ MEFs) (Komatsu et al., 2005) were incubated in Dulbecco's modified Eagle's minimum essential medium (DMEM; Sigma‐Aldrich, St. Louis, MO, USA) supplemented with 10% fetal bovine serum (FBS; Sigma) at 37°C with 5% CO_2_. EGFP‐Rab7, mCherry‐galectin‐3, EGFP‐galectin‐1, EGFP‐galectin‐8, EGFP‐galectin‐9s, and annexin A2‐EGFP stable transformants were described previously (Nishiumi et al., 2009). Transfection of HeLa cells with the expression clones was performed using FuGENE^®^ HD (Promega Corporation, Madison, WI, USA), in accordance with the manufacturer's instructions. Cotransfection with the expression clones, the pEXPR series described in the above section, and the ϕC31 integrase expression clone, pJTI^TM^ ϕC31 Int (Invitrogen), was performed at a mass ratio of 1:1. After culturing in a 6‐well plate for 24 hr, the approximate numbers of transiently transformed cells were determined by fluorescence microscopy and the cells were split into 10‐cm plates and cultured. After 48 hr of transfection, the cells were selected in medium containing 2–4 μg/ml of blasticidin S HCl (Sigma). Selection continued for 10–14 days, when the colonies became visible. The individual colonies were picked using a pipette tip and transferred to individual wells of a 24‐well plate. Surviving colonies were expanded for stock. EGFP‐galectin‐3 transient transfection was performed with FuGENE^®^ HD in accordance with the manufacturer's protocols.

### 2.8 Labeling of *U. parvum* cells

The *U. parvum* cells were cultured in 2 ml of UMCHs medium (Namba et al., 2010) at 37°C overnight. The color of the medium changed from yellow to red because of urea hydrolysis. The culture was separated by centrifugation at 15,000 × g for 15 min at 20°C. The pellets were suspended by washing twice with PBS and incubated with carbocyanine dye solution (Vybrant^TM^ DiI cell‐labeling solution DiI; Molecular Probes, Eugene, OR, USA). Five microliters of Vibrant DiI (dilution, 1:200) was added to *U. parvum* cells in 1 ml of PBS and incubated for 40 min at 37°C (Marques et al., 2010). The labeled *U. parvum* cells were separated by centrifugation for 15 min at 15,000 × g at 20°C, washed twice with PBS, gently suspended in 2% FBS in DMEM cell culture medium.

### 2.9 *U. parvum* infection of cultured cells

The HeLa, atg7^−/−^ and WT MEF cells, and several stable transformant cells were grown on poly‐L‐lysine‐coated glass coverslips (13 mm; Matsunami Glass Ind. Ltd., Osaka, Japan) to approximately 70% confluence (5 × 10^4^/ml) before they were infected with *U. parvum*. These cells were initially washed with PBS and then infected with DiI‐labeled *U. parvum* contained in 1 ml of DMEM with 2% FBS. The sets of inoculated cells were incubated at 37°C in a 5% CO_2_ atmosphere for 0, 0.5, 3, 6, or 24 hr.

### 2.10 Immunofluorescence and microscopy

After each period of infection, the bacterial suspension was gently removed and each well with a cell monolayer was washed three times with PBS. The infected HeLa cells were fixed in 4% paraformaldehyde in PBS for 15 min at room temperature, permeabilized with 0.1% Triton X‐100 for 10 min, and blocked with 2% bovine serum albumin (BSA) for 1 hr at room temperature. The cells were incubated with primary antibodies, including a rabbit polyclonal antibody against MBA (1:500), caveolin‐1 (caveolae marker; 1 μg/ml), the clathrin heavy chain (1 μg/ml) (Abcam, Cambridge, UK), anti‐human CD107a (LAMP‐1) purified (1 μg/ml) (eBioscience, San Diego, CA, USA), monoclonal anti‐LC3 (5 μg/ml) (MBL, Nagoya, Japan), or anti‐Rab11 (1 μg/ml) (BD Biosciences, New Jersey, USA), which were diluted with 2% BSA in PBS, at 4°C overnight**.** Next, the cells were stained with species‐specific Alexa 488‐conjugated secondary antibodies [Alexa Fluor 488F (ab′) 2 fragment of goat anti‐rabbit antibody, Alexa Fluor 488F (ab′) 2 fragment of goat anti‐mouse antibody, Alexa Fluor 648 goat anti‐mouse antibody, or Alexa Fluor 488 goat anti‐chicken antibody (Molecular Probes)] for 1 hr at room temperature. The lysosomes of HeLa cells infected with *U. parvum* were stained (LysoTracker Red, Invitrogen) for 30 min at 37°C. Then, the cells were washed with PBS and fixed. Actin and EEA1 were stained with anti‐Alexa 488 Phalloidin and anti‐EEA1‐Alexa Fluor 488 (10 μg/ml) (MBL). For immunofluorescence, a fluorescence microscope (Eclipse T*i*; Nikon Co., Tokyo, Japan) with a filter set for BEF/GFP/DsRed (86009; Chroma Technology Co., Bellow Falls, VT, USA) was used, and images were acquired using DS‐Qi2 (Nikon) or IXon^EM+^ (Andor Technology, Belfast, UK). The images were analyzed using NIS Elements software (Nikon). A confocal laser scanning microscope (FV‐5000; Olympus) equipped with a multiwavelength argon laser (458, 488, and 515 nm) and a HeNe laser with a wavelength of 543 nm was used.

### 2.11 Inhibition of *U. parvum* entry into HeLa cells by selective inhibitors

HeLa cells were plated on coverslips and incubated with 10 mM chlorpromazine (CPZ) dissolved in distilled water to make 1 mg/ml, 0.2 mM phenylarsine oxide (PAO) dissolved in dimethyl sulfoxide (DMSO) to make 50 mg/ml, or 10 mM of methyl‐β‐cyclodextrin (MβCD) (Sigma) dissolved in water to make 50 mg/ml stock solutions at 37°C for 30–45 min before infection. These cells were incubated with *U. parvum* for 30 min at 37°C in the presence of the drug. After 30 min, the cells were washed with PBS and fixed. The nocodazole treatment was applied 15 min after *U. parvum* infection involving incubation for 3 hr with 10 μM of nocodazole (Sigma) dissolved in DMSO to make a 10 mM stock solution.

### 2.12 Small interfering RNA (siRNA) transfection

The siRNA duplex was synthesized as a 21‐mer with UU overhangs (Ambion by Life Technologies Co.). The clathrin heavy chain target sequence was GGUUGCUCUUGUUACGGAU. Negative control siRNA sequences that did not target any gene product (Ambion) were also used. The siRNA duplex was resuspended in 50 μM nuclease‐free water before transfection. HeLa cells were seeded on coverslips with 4 μM of siRNA duplex (clathrin and negative control) and 5 μl of siPORT *NeoFX* (Ambion) in 300 μl of Opti‐MEM medium. Three days later, the cells were infected. Both infected and uninfected cell lysates were harvested for western blotting or immunofluorescence.

### 2.13 Western blot analysis

The cells were harvested after siRNA transfection or *U. parvum* infection and lysed in lysis buffer containing 20 mM Tris‐HCl (pH 7.5), 1% Nonidet P‐40, 0.1 M NH_4_SO_4_, 10% glycerol, and a protease inhibitor cocktail Set III (Calbiochem^®^; Merck KGaA, Darmstadt, Germany). Lysates were clarified by pipetting and rotated for 30 min at 4°C. Insoluble material was removed by centrifugation (15,000 × g). Equal amounts of protein were loaded onto a 7% or 12% SDS‐PAGE gel and transferred to polyvinylidene fluoride (PVDF) membranes (GE Healthcare Life Sciences, Little Chalfont, UK). Anti‐clathrin, polyclonal anti‐LC3 (MBL), anti‐CD9 antibody (Abcam), monoclonal ant‐Human CD63, annexin II (p36) antibody (BD Biosciences), MBA polyclonal antibody, and anti‐glyceraldehyde‐3‐phosphate dehydrogenase (GAPDH) monoclonal antibodies (antibody dilution, 1:1,000) (Ambion) were used to detect protein expression patterns. After incubation with horseradish peroxidase‐conjugated affinity‐purified anti‐mouse (Rockland Immunochemicals Inc., Gilbertsville, PA, USA) or goat anti‐rabbit (Zymed Laboratories, Inc., San Francisco, CA, USA) secondary antibodies, the blots were visualized using an enhanced chemiluminescence detection system (GE). The blots were also probed for GAPDH, which was used as a loading control.

### 2.14 Gentamicin invasion assay

The gentamicin invasion assay was performed to determine the rate of invasion of viable *U. parvum* into eukaryotic cells (Yavlovich, Tarshis, & Rottem, 2004). The *U. parvum* strains used in this study were tested for their susceptibility to the gentamicin concentration (Wako) utilized in this assay (200 μg/ml). WT MEF and *atg 7* ^−/−^ MEF cells were seeded in 6‐well plates. After 24 hr of incubation at 37°C in 5% CO_2_, the cell cultures were inoculated with *U. parvum* (7.5 × 10^4^ colony‐forming units [CFU]). The infected cells were incubated for 3 hr, washed three times with PBS, and incubated for additional 3 hr in DMEM containing 200 μg/ml gentamicin to eliminate the non‐internalized *U. parvum*. To determine intracellular survival at 6 hr, the plate was rinsed with 2% FBS DMEM and the medium was replaced with fresh medium without antibiotic. After 6 hr of inoculation, the *U. parvum*‐infected cells were rinsed twice with PBS, lysed, and the number of intracellular bacteria was determined. The infected cells were trypsinized and cultured in UMCHs (for *U. parvum*) medium for 48 hr. The culture medium was plated on UMCH agar plates for enumeration of the intracellular CFU of *U. parvum*. The intracellular bacterial assays were performed in quadruplicate and repeated three times.

### 2.15 *U. parvum* transmission assay

For *U. parvum* exocytosis experiments, EGFP‐annexin A2 was stably expressed in HeLa cells. At 3 hr postinfection, cells were treated with gentamicin (200 μg/ml). At 3 hr, extracellular *U. parvum* was removed by multiple washes. The supernatants of infected cells were collected 24 hr postinfection. The supernatants were added to WT HeLa cells and incubated for 24 hr. Then, cells were fixed with 4% PFA, permeabilized, subjected to staining with 4′,6‐diamidino‐2‐phenylindole (DAPI; Roche Diagnostic Co., Indianapolis, IN, USA), and observed using a fluorescence microscope (Nikon). The primary infection experiments were performed triplicates.

### 2.16 Isolation of exosomes

Exosomes were extracted from peripheral plasma using miRCURY^TM^ Exosome Isolation Kit (EXQON A/S, Vedbaek, Denmark), in accordance with the manufacturer's instructions. The precipitat from miRCURY^TM^ exosome pellets was lysed in 100 μl of resuspension buffer for western blot analysis.

### 2.17 Statistical analysis

To quantify the intracellular survival of *U. parvum* DNA and the suppression of *U. parvum* internalization using inhibitors, data were analyzed using one‐way analysis of variance with a Tukey–Kramer post hoc test. Student's *t* test was used to examine the number of infected/uninfected viable cells, colocalized signaling data, and the gentamicin invasion assay results. Differences were considered significant when *p* < .05. Error bars represent mean ± standard error of the mean (SEM).

## 3 RESULTS

### 3.1 Intracellular *U*. *parvum* is detected in fetal cells from chorioamnionitis placenta and mammalian cells

**Figure 1:**
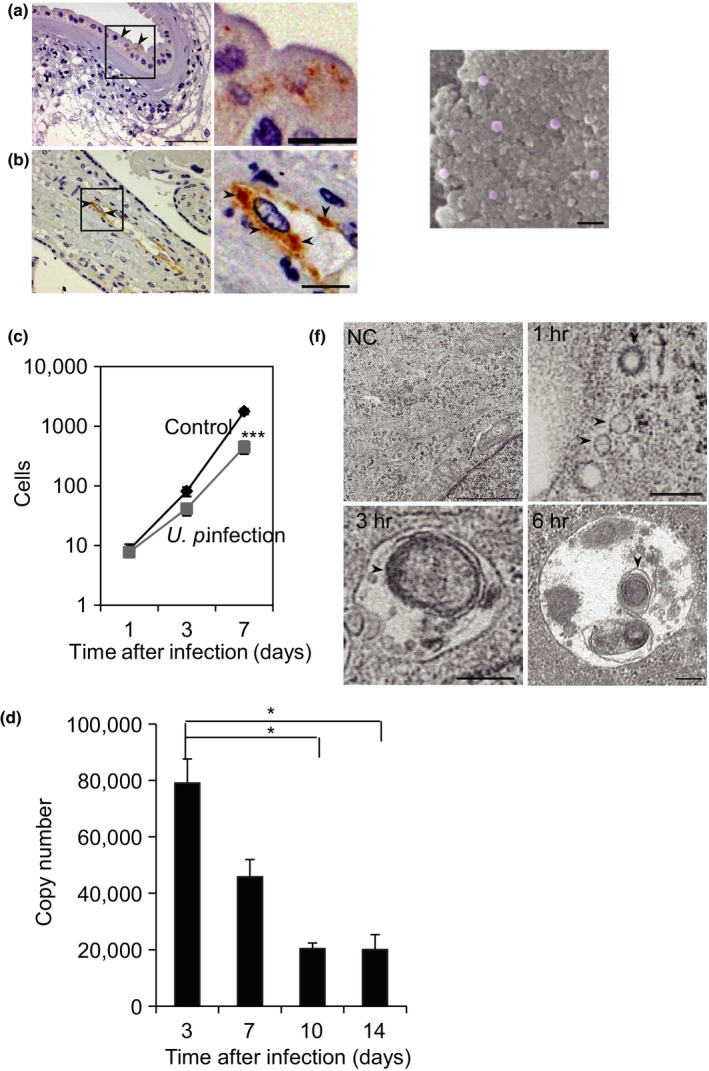
Distribution of *Ureaplasma parvum* in placenta and HeLa cells. *U. parvum* (arrowhead) was detected in the amniotic cell villous syncytiotrophoblasts (a) and endothelial cells (b). Scale bar: 50 μm. The boxed areas in (a) and (b) are enlarged in the right panel. Scale bar: 10 μm. (c) The number of viable cells infected by *U. parvum* compared with uninfected cells. ***p < .001 compared with the control group. (d) Intracellular survival of *U. parvum* in HeLa cells. After several days of infection, genomic DNA was isolated from cells. The expression of *U. parvum* DNA was determined by quantitative PCR. *p < .05 compared with the group infected for 3 days. (e) SEM analysis of a *U. parvum* colony. Purple colors indicate independent *U. parvum* cells. Scale bar: 500 nm. (f) Representative transmission electron microscopy images of HeLa cells uninfected (negative control; NC) or infected with *U. parvum* (arrowhead) for 1, 3, or 6 hr. NC scale bar: 1 μm, 1, 3, 6 hr scale bar: 200 nm

To determine the in vivo intracellular localization of *U. parvum* in chorioamnionitis placenta, we analyzed the results of immunohistochemical staining performed on such placentas that were positive for this bacterium. The MBA‐positive ureaplasma signals accumulated in the cytoplasm of amniotic cells, villous syncytiotrophoblasts, and endothelial cells (Figure 1a,b), while the negative (control) placentas did not show any positive signals (data not shown). Next, we cultivated infected HeLa cells for up to 7 days and performed a count of viable HeLa cells (Figure 1c). Three days after infection, no significant differences in the number of viable cells were observed between control and infected cultures. However, after 7 days, the number of *U. parvum*‐infected HeLa cells was significantly reduced, indicating that *U. parvum* caused growth retardation of HeLa cells. Next, the invasive abilities of *U. parvum* were determined by qPCR (Figure 1d). Internalization of *U. parvum* DNA into HeLa cells was confirmed and quantified in this assay. Compared with 3 days after infection, the number of intracellular *U. parvum* continued to decrease up to 10 days after infection and then reached a plateau.

To determine the actual size and shape of this microorganism, we analyzed infected *U. parvum* particles by SEM. We observed that these particles were regular and spheroid, with a mean diameter of 146.3 ± 45.3 nm, but with considerable variation (range 75–225 nm) (Figure 1e). This observation was then confirmed on the electron microscopy sections of host cellular membrane/vesicle‐bound *U. parvum* in HeLa cells by TEM (Figure 1f). After 1 hr of infection, *U. parvum* entered the cytoplasm and induced membrane fusion. Between 3 and 6 hr of infection, the intracellular *U. parvum* formed a phagolysosome.

### 3.2 *U. parvum* invasion of mammalian cells requires clathrin‐dependent endocytosis

**Figure 2:**
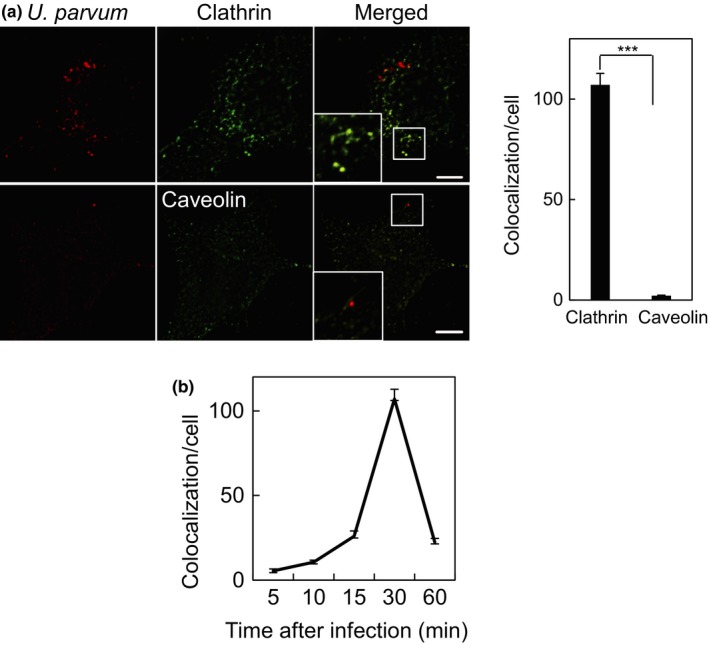
The internalized *Ureaplasma parvum* colocalized with clathrin in HeLa cells. (a) *U. parvum* entered the cells through clathrin‐ or caveolin‐dependent endocytosis. HeLa cells were uninfected or infected by *U. parvum* (red) for 0.5 hr at 37°C and merged with clathrin (top green) or caveolin (bottom green). Scale bar: 10 μm. The number of signals of *U. parvum* colocalization with clathrin (left bar) or caveolin (right bar) per infected HeLa cell (mean ± SEM,* n* = 200 cells; ***p < .0001 compared with caveolin). (b) The number of signals of *U. parvum* colocalization with clathrin per cell for various indicated times (mean ± SEM,* n* = 200 cells)

To provide further information on *U. parvum* entry, the colocalization of *U. parvum* with endocytosis‐related molecules, clathrin heavy chain and caveolin‐1, was observed. After 30 min of infection, colocalization of *U.  parvum* and clathrin was observed as a spotted pattern in the perinuclear region (Figure 2a). In contrast, the caveolin‐1 protein did not colocalized with *U. parvum* (Figure 2a). We observed the colocalization of clathrin and *U. parvum* 5 min after infection (Figure 2b). The colocalization of clathrin and *U. parvum* showed rapid changes. It was detected at a very early stage of at least 5 min, peaked at 30 min, and then decreased after 1 hr of infection. These results demonstrated that *U. parvum* was internalized into the host cell through a clathrin‐dependent pathway.

**Figure 3:**
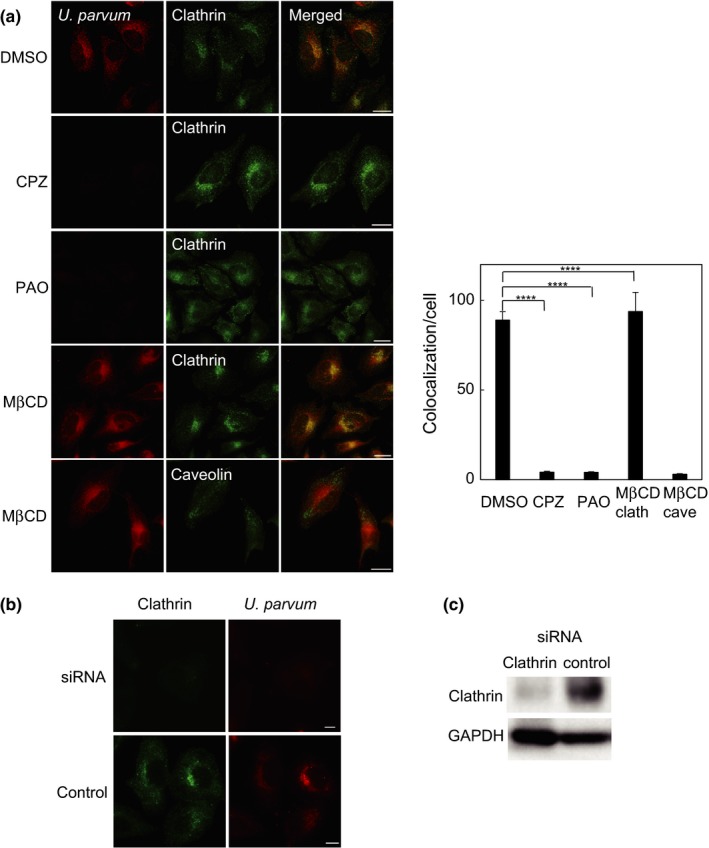
The internalization of *Ureaplasma parvum* was suppressed by clathrin inhibitors and siRNA. (a) Fluorescent images after CPZ, PAO, and MβCD treatment and DiI‐labeled *U. parvum* (red) infection for 0.5 hr at 37°C. Clathrin or caveolin (green) was also stained and merged. Scale bar: 10 mm. The internalized signal numbers of *U. parvum* and clathrin or caveolin per cell were quantified (mean ± SEM,* n* = 200 cells; ***p < .0001 compared with DMSO). (b) HeLa cells were transfected with clathrin siRNA or control siRNA for 72 hr and then infected with *U. parvum* for 0.5 hr at 37°C. (c) The protein expression level of clathrin was detected by western blot after siRNA transfection (left lane) or control treatment (right lane). GAPDH was measured as a loading control

We next investigated the role of clathrin in *U. parvum* infection. CPZ is a commonly used inhibitor of clathrin‐coated pit formation by the reverse translocation of clathrin and its accessory proteins from the plasma membrane to intracellular vesicles. PAO is another inhibitor of clathrin‐dependent endocytosis. In a control experiment, as shown in Figure 3a, U*. parvum* localized around the nucleus of untreated DMSO HeLa cells 3 hr after infection. In contrast, CPZ and PAO treatments completely blocked *U. parvum* invasion of HeLa cells without the loss of clathrin itself (Figure 3a). When HeLa cells were treated with MβCD, a caveolin‐dependent endocytosis inhibitor, the internalization of *U. parvum* into HeLa cells was not inhibited. Furthermore, we used a well‐characterized siRNA approach to knockdown the gene expression of the clathrin heavy chain in the *U. parvum* invasion assay. At 72 hr after transfection, siRNA‐treated cells showed very low levels of clathrin both in cellular observations (Figure 3b) and through western blotting (Figure 3c). After infection, *U. parvum* also appeared in the perinuclear region in the control cells, whereas siRNA transfection completely blocked *U. parvum* entry. These results suggested that both clathrin itself and the clathrin‐related pathway were required for the entry of *U. parvum* into HeLa cells.

### 3.3 *U. parvum* is trafficked along microtubules and enters endosomes

**Figure 4:**
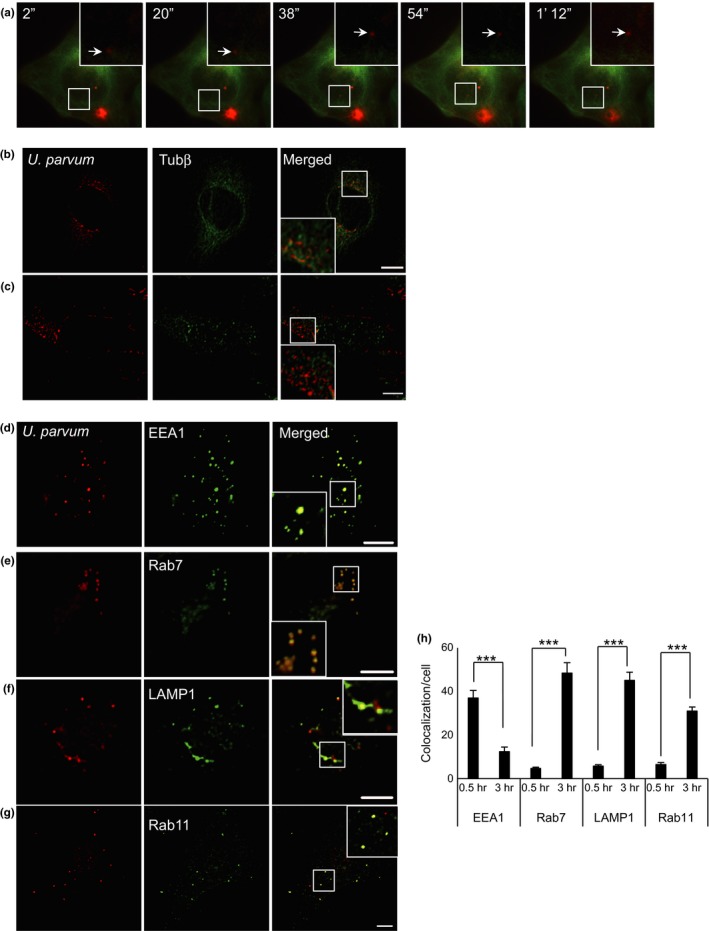
Colocalization of *Ureaplasma parvum* with endosome markers and transfer along the microtubules. (a) HeLa cells stably expressing tau‐YFP were infected with DiI‐labeled *U. parvum* for 2 hr. After infection, images were acquired at 2‐s intervals. The movement of *U. parvum* from the cell surface to the perinuclear area is shown. HeLa cells were infected with *U. parvum* for 3 hr, used as a control (b) or subjected to nocodazole treatment (c). The cells were fixed and stained with anti‐β‐tubulin antibody, followed by Alexa 648 secondary antibody. Scale bar: 10 μm. (d) HeLa cells were infected with *U. parvum* for 0.5 hr at 37°C. EEA1 was detected with anti‐EEA1 antibody. Scale bar: 10 μm. (e) The HeLa cells stably expressing GFP‐Rab7 were infected with *U. parvum* for 3 hr. The cells were visualized by fluorescence microscopy. Scale bar: 10 μm. (f) The HeLa cells were infected with *U. parvum* for 3 hr at 37°C. LAMP‐1 (green) was detected with anti‐LAMP‐1 antibody. Scale bar: 10 μm. (g) HeLa cells were infected with *U. parvum* for 3 hr at 37°C. Rab11 was detected with anti‐Rab11 antibody, followed by Alexa 488 secondary antibody. Scale bar: 10 μm. (h) Quantification of *U. parvum* colocalized with EEA1, Rab7, and LAMP‐1 per cell after 0.5 and 3 hr of infection (mean ± SEM,* n* = 200 cells; ***p < .001 compared with 0.5 hr)

We prepared a stable transformant of HeLa cells expressing tau protein‐fused EYFP to follow the direct involvement of microtubules during DiI‐labeled *U. parvum* trafficking in live cells. Cells expressing tau‐EYFP were infected with DiI‐labeled *U. parvum* and transport of *U. parvum* along microtubules was followed by time‐lapse fluorescence microscopy (Figure 4a). As shown in Figure 4b, U*. parvum* infected HeLa cells, which were also subjected to nocodazole treatment for 3 hr (Figure 4c). The results showed changes in the intracellular localization of *U. parvum*. It accumulated in the perinuclear region in control cells, while upon nocodazole treatment, *U. parvum* spread throughout the cells (Figure 4b,c). These results suggested that *U. parvum* was transported to the cytoplasm along microtubules. Endosomes are dynamic membrane systems involved in transport within the cells and they receive endocytosed molecules, sorting these for degradation or recycling them back to the cell surface. We demonstrated that *U. parvum* and EEA1 colocalization 30 min after infection (Figure 4d). Furthermore, the late endosome marker Rab7 (Figure 4e), LAMP‐1 (Figure 4f), and recycling endosome marker Rab11 (Figure 4g) colocalized with *U. parvum* 3 hr after infection. Figure 4h shows the colocalization of *U. parvum* and early to late endosome markers at infection times from 30 min to 3 hr. A large number of intracellular *U. parvum* colocalized with EEA1 30 min after infection, but this colocalization decreased after 3 hr. In contrast, colocalization of late endosome marker and *U. parvum* was negligible 30 min after infection, but increased 3 hr after infection. These findings demonstrated that *U. parvum* enters the cells through the clathrin‐mediated pathway and the early to late or recycling endosome pathway.

### 3.4 Intracellular interactions of galectins with *U. parvum*

**Figure 5:**
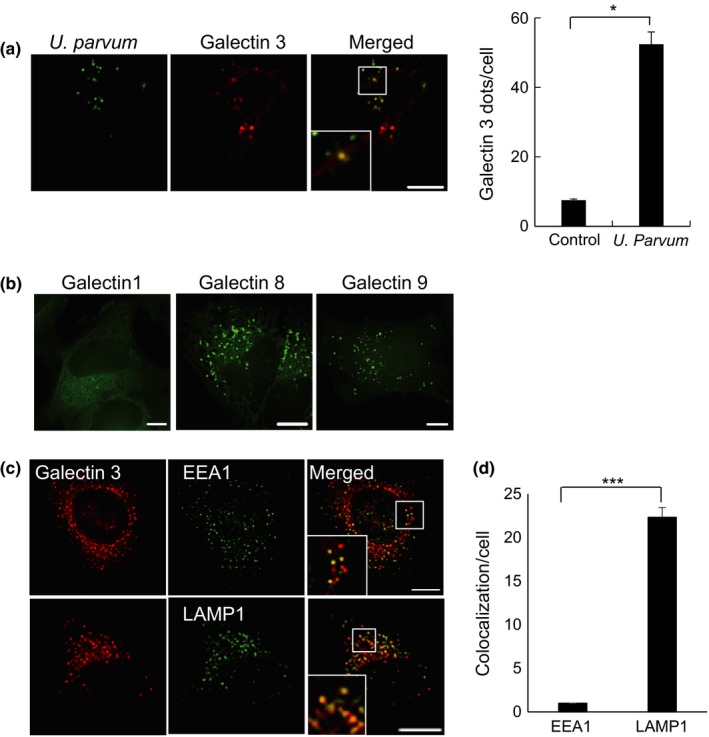
*Ureaplasma parvum* induced galectin‐3 accumulation around bacteria. (a) HeLa cells stably expressing mCherry‐galectin‐3 were infected with *U. parvum* for 0.5 hr at 37°C. Scale bar: 10 μm. Quantification of *U. parvum* colocalized with accumulated galectin‐3 signals is indicated in the middle panel. At least 200 cells were counted for the control and also for *U. parvum*‐infected cells (mean ± SEM, At least 200 puncta; *p < .05 compared with control). (b) HeLa cells that stably express EGFP‐galectin‐1, EGFP‐galectin‐8, and EGFP‐galectin‐9s were infected with unlabeled *U. parvum* for 3 hr. Scale bar: 10 μm. (c) HeLa cells that stably expressed mCherry‐galectin‐3 were infected with *U. parvum* for 3 hr. The cells were then fixed and stained with anti‐EEA1 and ‐LAMP‐1 (green) antibodies, followed by Alexa 488 secondary antibody. Scale bar: 10 μm. (d) Quantification of accumulated galectin‐3 signals colocalized with markers, as indicated in the panel (mean ± SEM,* n* = 200 puncta; ***p < .0001 compared with EEA1)

Intracellular galectin‐3 is a sensor of the vacuole that contains damaged bacteria for autophagosome degradation. After infection of HeLa cells with *U. parvum* for 3 hr and massive accumulation of a stable transformant of mCherry‐galectin‐3 its signals were observed in the vicinity of intracellular *U. parvum* (Figure 5a, infection and graph). To eliminate the possibility of galectin‐3 overexpression causing this accumulation stable transformant of EGFP‐galectin‐1, EGFP‐galectin‐8, and EGFP‐galectin‐9s in HeLa cells was established. Similar accumulations of galectin‐3, ‐8 and ‐9 were also observed during *U. parvum* infection of HeLa cells expressing EGFP‐galectin‐8 and EGFP‐galectin‐9s (Figure 5b). These findings prompted us to investigate the process of autophagy of *U. parvum*. Additional experiments revealed correlations between galectin‐3, EEA1, and LAMP‐1 with *U. parvum* in HeLa cells expressing mCherry‐galectin‐3. After infection of mCherry‐galectin‐3 stable transformant cells with *U. parvum* for 3 hr, galectin‐3 signals colocalized with LAMP‐1 (Figure 5c,d). Therefore, *U. parvum*‐induced galectin‐3‐positive endosomal damage occurred at the late endosome and the lysosome.

### 3.5 Involvement of *U. parvum*‐induced autophagosomes

**Figure 6:**
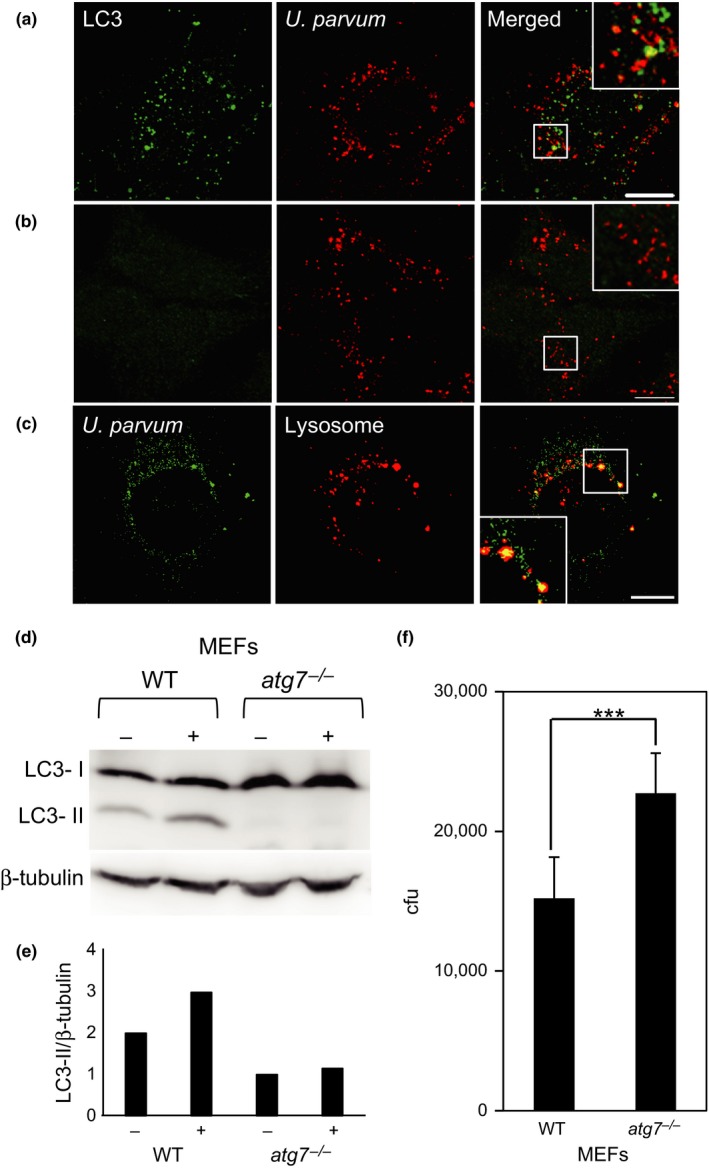
Effect of autophagy in agonist‐treated *Ureaplasma parvum*‐infected MEF cells. (a) Control MEFs and (b) *atg7* ^−/−^ MEFs were infected with *U. parvum* for 3 hr. LC3 was detected with anti‐LC3 antibody. Scale bar: 10 μm. (c) HeLa cells were infected with *U. parvum* for 3 hr. Lysosomes were detected with LysoTracker. Scale bar: 10 μm. (d) Control MEFs or *atg7* ^−/−^ MEFs in a 10‐cm dish were infected with *U. parvum* for 3 hr and LC3 western blotting was conducted. β‐tubulin was measured as a loading control. (e) Quantification of LC3‐II to β‐tubulin ratio in (d). (f) Comparison of viable, internalized *U. parvum* CFU of control WT or *atg7* ^−/−^ MEF cells by gentamicin invasion assay (mean ± SEM,* n* = 6; ***p < .001 compared with WT)

LC3 is a canonical autophagosome marker. To evaluate *U. parvum‐*induced autophagosomes, we used mouse embryo fibroblasts (MEFs) and *atg7* ^−/−^ MEFs. The effect of *U. parvum* on LC3 distribution was analyzed further by immunostaining and western blotting for endogenous LC3 in MEFs and *atg7* ^−/−^ MEFs. A number of LC3 puncta were observed in wild‐type (WT) MEFs and these increased markedly in response to *U. parvum* infection (Figure 6a). When *atg7* ^−/−^ cells were used, LC3 puncta were not generated following *U. parvum* infection (Figure 6b). The punctate formations of the autophagic marker LC3 colocalized with *U. parvum* in the infected WT MEF cells compared with those the *atg7* ^−/−^ MEF cells. In the *U. parvum*‐infected MEFs and *atg7* ^−/−^ MEFs, LC3 appeared to be recruited to the autophagosome membranes mainly in the perinuclear region. These signals colocalized with *U. parvum*, at least indicating the presence of autophagy‐mediated ureaplasma clearance. Moreover, the *U. parvum*‐infected HeLa cells incubated with LysoTracker Red dye showed the characteristic red fluorescence of lysosomes (Figure 6c). These findings also suggested that colocalization of part of the incorporated *U. parvum* and the lysosome had occurred and that the intracellular *U. parvum* degradation process was mediated by autophagy. We also investigated whether *U. parvum* induced lipidation of LC3, which is essential for the translocation of LC3 from the cytosol to autophagosomes. The lipidated form of LC3 (LC3‐II) has increased mobility on SDS‐PAGE relative to its unlipidated form. Figure 6d shows a western blot using an antibody against LC3. The ratio between LC3‐II and β‐tubulin was increased in WT MEF after *U. parvum* infection for 3 hr (Figure 6e). These results suggested that LC3 is an important component for in *U. parvum* degradation within the host cell. The results from the gentamicin invasion assay showed that *U. parvum* was increased more in *atg7* ^−/−^ MEF cells compared with MEF cells (Figure 6f). These results suggested that the autophagic machinery is involved in *U. parvum* degradation.

### 3.6 Exosomes derived from *U. parvum*‐infected HeLa cells contain ureaplasma

**Figure 7:**
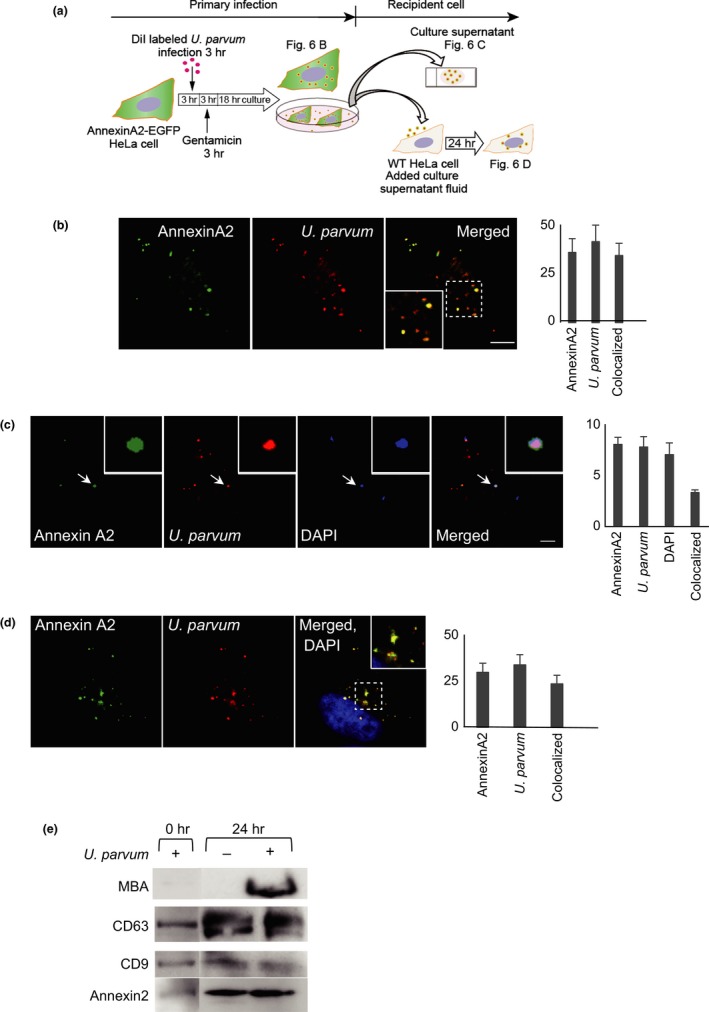
*Ureaplasma parvum* localized to the recycling endosome and exocytosis with Annexin A2. (a) Schematic representation of infection experiments. (b) HeLa cells stably expressing EGFP‐annexin A2 were infected with DiI‐labeled *U. parvum* for 24 hr. Scale bar: 10 μm. (c) The supernatants from the infected cells (b) were collected. The fluorescence of DiI labeled *U. parvum*, EGFP‐annexin A2, and DAPI were detected. White arrow indicates annexin A2‐coated *U. parvum*. Scale bar: 10 μm. (d) The supernatants from infected cells (c) were supplemented with normal HeLa cells and cultured for 24 hr. Scale bar: 10 μm. (e) The detection of MBA, exosome marker (CD9, CD63), and annexin II in exosomes prepared from *U. parvum* infected/or uninfected HeLa cell supernatants by western blot

To investigate the functional role of exosomes in the transmission of infection, exosomes were isolated from *U. parvum*‐infected cells and incubated with WT HeLa cells, as outlined in Figure 7a. As shown in Figure 7b, after the infection of EGFP‐annexin A2 stable transformant cells with DiI‐labeled *U. parvum* for 24 hr, annexin A2 signals colocalized with *U. parvum*. Fluorescence microscope imaging confirmed the exosome preparations (Figure 7c). Annexin A2 and DiI‐labeled *U. parvum* colocalized in exosomes isolated from *U. parvum*‐infected EGFP‐annexin A2 stable transformant HeLa cells. One day after exposure to *U. parvum*‐positive exosomes, WT HeLa cells were observed by fluorescence microscopy (Figure 7d). Finally, exosomes were prepared from the supernatants of *U. parvum*‐infected HeLa cells and their protein content was analyzed. As shown in Figure 7e, the concentrated exosome preparations were specifically enriched in the exosome markers CD9, CD63, annexin A2, and also ureaplasmal MBA. In addition, we showed that at the time point of 0 hr of the infection assay *(U. parvum* +), the exosome preparation did not contain free *U. parvum*. Annexin A2 plays a key role in membrane vesicle trafficking. These results indicate that the *U. parvum*‐positive exosomes were able to establish a secondary infection of WT HeLa cells, confirming that the exosome pathway results in productive infection.

## 4 DISCUSSION

The class *Mollicutes* comprises some of the smallest and simplest self‐replicating bacteria. Because *Ureaplasma* spp. have an extremely small genome, these organisms have limited metabolic options for replication and survival. Such limitation may obstruct the establishment of genetically engineered knockout and transgenic ureaplasma. Because of their limited biosynthetic capabilities, most mycoplasmas are parasites exhibiting strict host and tissue specificities. *Mycoplasma spp*. and *U. diversum* (Marques et al., 2010) attach to or enter host cells, wherein they multiply and survive for a long period. These microorganisms have evolved key molecular mechanisms that can subvert the host immune response, enabling transfer, and colonization into a new host (Hopfe, Deenen, Degrandi, Kohrer, & Henrich, 2013; Rottem, 2003).

**Figure 8:**
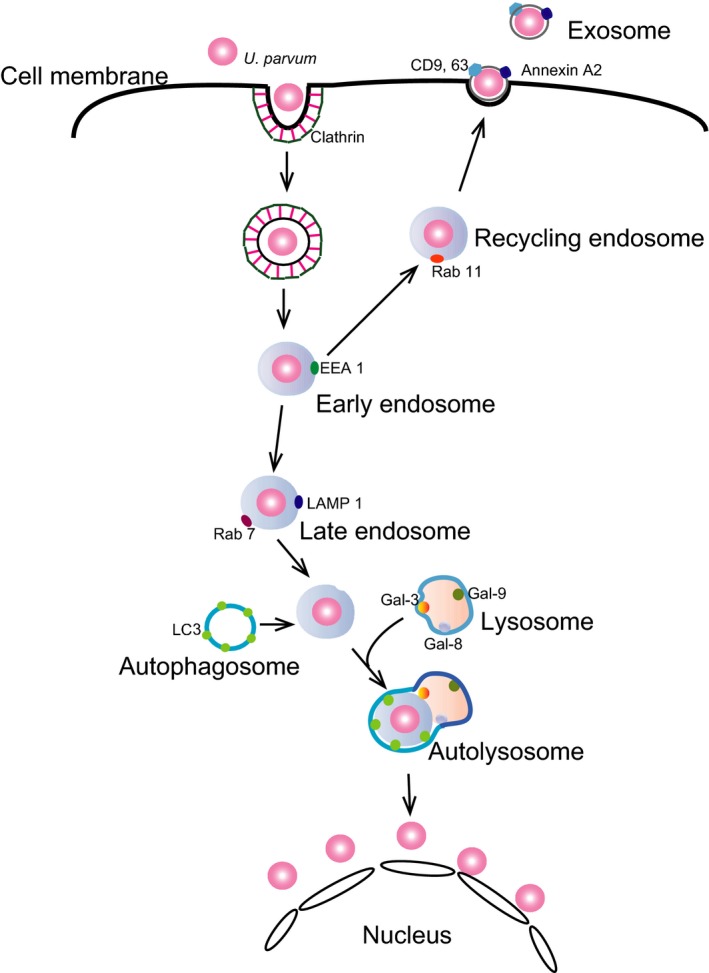
Proposed model of *Ureaplasma parvum* infection mechanism. This model shows that *U. parvum* enters HeLa cells through clathrin‐dependent endocytosis and is transported to early endosomes. From there, it can be transported through recycling endosomes, exported through exocytosis, or transported through late endosomes where the host cell responds by neutralizing it through lysosomal degradation or autophagic machinery. However, *U. parvum* can evade these mechanisms and localize perinuclearly

In this study, *U. parvum* displayed phenotypes of intracellular bacteria for at least 7 days in HeLa cells. Details of the mechanisms underlying *U. parvum* infection are unknown. Here, we first demonstrated that clathrin‐dependent endocytosis was required for *U. parvum* infection in these cells, similar to the viral entry pathway (Figure 8) (Bhattacharyya et al., 2010; Wang, Rothberg, & Anderson, 1993). The size of *U. parvum* used in this study varied from 75 to 225 nm. Its size is similar to the molecular diameter of clathrin‐mediated endocytic vesicles (85–110 nm). We demonstrated *U. parvum* entry into HeLa cells by inhibiting clathrin‐dependent endocytosis via CPZ, PAO, and siRNA. CPZ inhibits calmodulin (Marshak, Lukas, & Watterson, 1985; Wrenn, Katoh, Schatzman, & Kuo, 1981), which can bind to the phospholipid components in the plasma membrane of endothelial cells (Hueck, Hollweg, Schmid‐Schonbein, & Artmann, 2000). We showed that CPZ potently inhibits *U. parvum* infection and that clathrin‐dependent endocytosis is necessary for infection.

Generally, during clathrin‐dependent endocytosis, incoming viruses are transported together with their receptors from the plasma membrane into early and late endosomes. Rab5, Rab7, LAMP‐1, and Rab11 are associated with compartments along the endocytic pathway; Rab5 was detected at the cytoplasmic surface of both the plasma membrane and early endosomes, whereas Rab7 and LAMP‐1 were associated with late endosomes and Rab 11 with recycling endosomes (Figure 8). Rab7 plays an important role in the fusion and transport of cargo from late endosomes to lysosomes (Bottger, Nagelkerken, & van der Sluijs, 1996; Bucci, Thomsen, Nicoziani, McCarthy, & van Deurs, 2000; Meresse, Gorvel, & Chavrier, 1995; Papini et al., 1997; Schimmoller & Riezman, 1993). We showed that the entry route for *U. parvum* into HeLa cells involves early to late endosomes and recycling endosomes. As expected, the intracellular transport of *U. parvum* toward the perinuclear region is associated with the microtubules.

Several previous studies identified a correlation between galectin‐3 and the endocytic pathway via the former's interaction with two lysosomal/late endosomal proteins, LAMP‐1 and LAMP‐2 (Dong & Hughes, 1997; Sarafian et al., 1998). LC3‐positive autophagosomes colocalized with galectin‐3, ubiquitin, and p62/SQSTM1 (Chen et al., 2014). In this study, we showed galectin‐3 accumulation proximal to the invading bacteria. Moreover, the partial targeting of galectin‐3 to the LAMP‐1‐positive endosomes may explain the minor overlap of *U. parvum*‐infected cells. Galectin‐8 can target the vacuole containing damaged bacteria for autophagosome degradation; therefore, it is considered a danger receptor that restricts intracellular bacterial proliferation (Thurston, Wandel, von Muhlinen, Foeglein, & Randow, 2012). Furthermore, we found that galectin‐8 accumulated in cells infected with *U. parvum*. These observations indicated the possibility that *U. parvum* is a vesicle‐damaging pathogen. However, future studies will be needed to clarify this issue.

Although some bacteria are killed by autophagy, others can evade or even exploit autophagy to cause diseases. For example, *Shigella flexneri* can evade autophagic capture in the cytosol (Ogawa et al., 2005). This bacterium enters host cells and escapes from the phagosome into the cytosol (Cemma & Brumell, 2012). Autophagy is a dynamic process consisting of the formation and fusion of membrane compartments. Here, we report that the induction of autophagy occurred in the initial stage after infection (3 hr) with *U. parvum*. To understand how autophagy plays a role during *U. parvum* infection, we analyzed the localization of autophagosome markers. We observed the locations of LC3 and *U. parvum* by fluorescence microscopy, which demonstrated that they colocalized with each other (Figure 8). The degradation of *U. parvum* in the phagosome is, at least in part, mediated by autophagy in MEF cells.

It is known that EGFP‐tagged galectin‐3 is recruited to the bacterial entry site within seconds after vacuolar rupture and targets the disassembling membranes surrounding the bacterium (Paz et al., 2010). Furthermore, the role of galectin‐8 in targeting bacterially damaged vesicles for autophagy has been convincingly described. Therefore, galectins may function in a manner similar to antimicrobial peptides and play an important role in innate immunity through this mechanism.

Exosomes are vehicles established for the shuttling of proteins, mRNA, and miRNA between cells (Valadi et al., 2007); as such, they play an important role in many biological processes (Duijvesz, Luider, Bangma, & Jenster, 2011; Thery, Zitvogel, & Amigorena, 2002). Although a role of exosomes in the shuttling of infectious agents between cells has been postulated, this has still not been extensively demonstrated. We showed that *U. parvum* infection can be transmitted by exosomes between HeLa cells and can establish a productive infection (Figure 7). In general, the mechanism of uptake of exosomes by cells is not fully understood. Further research is required to determine the pathway involved in the uptake of exosomes and *U. parvum*. Our data indicated that urease‐producing *Ureaplasma* may survive in low‐pH environments such as in the lysosome. Although the precise escape mechanism remains unknown, *U. parvum* appeared to induce significant endosomal–lysosomal damage. Cytoplasmic *U. parvum* was recognized by galectin‐3, a component of the innate immune system; this may form the basis for its interaction with autophagosomes. The precise molecular basis behind the mechanism of ureaplasmal host cell membrane damage should be elucidated in the future.

It is widely accepted that bacteria ascending from the vagina after initial colonization are the main cause of preterm birth (Goldenberg, Hauth, & Andrews, 2000). *Ureaplasma* spp. is one of the pathogenic organisms most commonly detected in the amniotic fluid (Kacerovsky et al., 2013; Ueno et al., 2015), but its mechanism of tissue invasion remains unknown. Thus, in this study, we focused on clarifying the mechanisms underlying the cellular invasiveness of *Ureaplasma* spp. and found that it has the potential to survive intracellularly by escaping lysosome degradation and autophagic elimination. We further revealed the in vivo intracellular localization of *Ureaplasma* spp. in fetal‐derived cells of infected placenta. We thus clarified the mechanisms of *Ureaplasma* spp. invasion; however, because *Ureaplasma* spp. are commonly found in the vagina of many women in reproductive age, the host factor responsible for preterm birth remains to be determined.

## CONFLICT OF INTEREST

None declared.
